# Value Assessment of Health Losses Caused by PM_2.5_ Pollution in Cities of Atmospheric Pollution Transmission Channel in the Beijing–Tianjin–Hebei Region, China

**DOI:** 10.3390/ijerph16061012

**Published:** 2019-03-20

**Authors:** Zhixiang Xie, Yang Li, Yaochen Qin, Peijun Rong

**Affiliations:** 1 College of Environment and Planning, Henan University, Kaifeng 475004, China; zhixiang1108@163.com (Z.X.); liyanghenu@163.com (Y.L.); 2 Key Laboratory of Geospatial Technology for Middle and Lower Yellow River Regions, Henan University, Kaifeng 475004, China; 3 College of Tourism and Exhibition, Henan University of Economics and Law, Zhengzhou 450046, China; rongpeijun@126.com

**Keywords:** health losses, value assessment, exposure–response coefficient, atmospheric pollution transmission channel in the Beijing–Tianjin–Hebei region

## Abstract

A set of exposure–response coefficients between fine particulate matter (PM_2.5_) pollution and different health endpoints were determined through the meta-analysis method based on 2254 studies collected from the Web of Science database. With data including remotely-sensed PM_2.5_ concentration, demographic data, health data, and survey data, a Poisson regression model was used to assess the health losses and their economic value caused by PM_2.5_ pollution in cities of atmospheric pollution transmission channel in the Beijing–Tianjin–Hebei region, China. The results showed the following: (1) Significant exposure–response relationships existed between PM_2.5_ pollution and a set of health endpoints, including all-cause death, death from circulatory disease, death from respiratory disease, death from lung cancer, hospitalization for circulatory disease, hospitalization for respiratory disease, and outpatient emergency treatment. Each increase of 10 μg/m^3^ in PM_2.5_ concentration led to an increase of 5.69% (95% CI (confidence interval): 4.12%, 7.85%), 6.88% (95% CI: 4.94%, 9.58%), 4.71% (95% CI: 2.93%, 7.57%), 9.53% (95% CI: 6.84%, 13.28%), 5.33% (95% CI: 3.90%, 7.27%), 5.50% (95% CI: 4.09%, 7.38%), and 6.35% (95% CI: 4.71%, 8.56%) for above-mentioned health endpoints, respectively. (2) PM_2.5_ pollution posed a serious threat to residents’ health. In 2016, the number of deaths, hospitalizations, and outpatient emergency visits induced by PM_2.5_ pollution in cities of atmospheric pollution transmission channel in the Beijing–Tianjin–Hebei region reached 309,643, 1,867,240, and 47,655,405, respectively, accounting for 28.36%, 27.02% and 30.13% of the total number of deaths, hospitalizations, and outpatient emergency visits, respectively. (3) The economic value of health losses due to PM_2.5_ pollution in the study area was approximately $28.1 billion, accounting for 1.52% of the gross domestic product. The economic value of health losses was higher in Beijing, Tianjin, Shijiazhuang, Zhengzhou, Handan, Baoding, and Cangzhou, but lower in Taiyuan, Yangquan, Changzhi, Jincheng, and Hebi.

## 1. Introduction

Since the 1930s, there have been several famous atmospheric pollution events around the world, such as the Meuse Valley fog in Belgium, the photochemistry smoke event of Los Angeles, USA, the London smog episode in the United Kingdom and the asthma in Japan, which not only have caused huge economic losses, but also cost the health and lives of residents [1]. Among various atmospheric pollutants, fine particulate matter (PM_2.5_) is widely considered as the culprit causing health losses of residents due to its characteristics of small particle size, remote transmission distance, long duration, richness in toxic substances, and the ability to destroy the body’s blood circulation system [2,3,4]. According to the global burden of disease study, PM_2.5_ pollution caused approximately 4.2 million deaths, leading to 103 million losses of disability adjusted life years (DALYs) in 2015, and PM_2.5_ has become the fifth leading cause of death [5,6]. With the rapid progress of industrialization and urbanization, China is also faced with a severe atmospheric pollution problem. Haze weather, which is typically represented by PM_2.5_, occurs with a high frequency, a wide range and an unprecedented degree of harm, and it has become an important issue affecting China’s environmental quality, residents’ health and sustainable social development. It is estimated that PM_2.5_ pollution in Beijing city caused more than 20,000 deaths and more than 1 million people to fall ill, resulting in direct economic losses of approximately $147.62 million in 2013 [7]. Therefore, it is of great significance to evaluate the health losses of residents induced by PM_2.5_ pollution and to calculate their economic value for promoting the prevention of atmospheric pollution and implementing China’s health strategy.

Non-Chinese scholars’ studies on the health losses of atmospheric pollutants can be traced back to the 1960s. Ridker [8] estimated that the economic value of residents’ health losses induced by SO_2_ pollution in the United States in 1958 was $80.2 billion using the human capital method, which became the beginning of quantitative assessment of the health effect of atmospheric pollutants. Dockery et al. [9] used the cohort study method to track the PM_2.5_ concentration and the health status of more than 8,000 residents in six cities in the United States, and they found that the death risk would increase by approximately 14% for every 10 μg/m^3^ increase in PM_2.5_ concentration. Based on the data of PM_2.5_ concentration and health information of 552,138 adults in 151 large cities in the United States from 1982 to 1989, Pope et al. [10] revealed that every 10 μg/m^3^ increase in PM_2.5_ concentration resulted in a 4.0% increase in all-cause mortality and an 8.0% increase in cardiopulmonary disease mortality, respectively. Katanoda et al. [11] observed the health effects of concentration changes in various air pollutants in Japan on 63,520 respondents from 1983 to 1985, and they found that the increase in PM_2.5_, SO_2_, and NO_2_ concentration lead to the increase in the death risk by lung cancer among residents. Among them, the death risk of lung cancer increased by 1.24% (95% CI: 1.12%, 1.37%) for every 10 μg/m^3^ increase in PM_2.5_ concentration; the death risk by lung cancer increased by 1.26% (95% CI: 1.07%, 1.48%) and 1.17% (95% CI: 1.10%, 1.26%) for each 10 ppb increase in SO_2_ and NO_2_ concentration. The study Atmospheric Pollution and Health: a European Approach (APHEA) showed that for every 10 μg/m^3^ increase in PM_10_ concentration, the hospitalization risk of asthma and chronic obstructive pulmonary disease (COPD) increased by 1.0% (95% CI: 0.4%, 1.5%) and by 0.5% (95% CI: 0.2%, 0.8%) for cardiovascular disease among people who were over 65 years old [12]. The increase in epidemiological cases makes it possible to obtain the exposure–response coefficient outside the case area by using the meta-analysis method, which also lays a solid foundation for accounting for the health losses due to air pollutants. Seethaler et al. [13] used the willingness-to-pay method to evaluate the economic value of health losses induced by PM_10_ pollution in Austria, France and Sweden in 1996 as €27 billion, accounting for 1.7% of their GDP in the same year. Quah et al. [14] used the environmental damage function and the dose–response method to estimate that the economic value of health losses caused by PM_10_ pollution in Singapore in 1999 was $3.662 billion, accounting for 4.31% of the GDP of that year. Chinese scholars started paying attention to the economic value of health losses caused by atmospheric pollution more recently; Guo et al. [15] calculated the harm to human health due to SO_2_ pollution in China in 1985 with the help of human capital approach, and they concluded that the health losses of residents were $1.28 billion. Chen et al. [16] estimated the health losses induced by PM_10_ pollution and their economic value in 113 Chinese cities in 2006, and they found that PM_10_ pollution caused 299,700 deaths, 254,900 hospitalizations and 7,625,100 medical outpatient visits, with an economic value of $43.72 billion. Based on the calculation of the health losses of residents caused by PM_2.5_ pollution in the Beijing–Tianjin–Hebei region, Huang et al. [17] believed that the health benefits of controlling PM_2.5_ pollution in this region could reach $28.36 billion. In addition, some scholars believed that the above methods only included the direct value assessment of health losses, so they advocated including the labor losses and medical expenses caused by atmospheric pollution in the model, so as to estimate the indirect impact of atmospheric pollution on the macro economy [18,19]. Through reviewing relevant literature, it is found that the current research has the following problems: (1) The exposure–response coefficient is the key to calculating the health and economic losses of residents induced by PM_2.5_ pollution. Due to the lack of epidemiological research cases in China, existing studies mostly refer to the results of the exposure–response coefficient in other countries [20]. However, the difference in PM_2.5_ pollution between China and other countries determines that using only foreign exposure–response coefficients will lead to a deviation of the evaluation results. (2) The current value assessment of health losses induced by PM_2.5_ pollution focuses on the death effect, and it pays insufficient attention to the pathogenic effect of PM_2.5_ pollution [21,22]. In fact, PM_2.5_ pollution not only increases the number of deaths, but also greatly increases the number of patients at different health endpoints. Therefore, measuring health losses from the perspective of the death effect is a one-sided approach. (3) The statistical life value determined by using the willingness-to-pay method is the main method that measures the unit value of death loss; however, most of the existing studies directly refer to the investigation of the residents’ willingness to pay for reducing atmospheric pollution health hazards carried out in the early stage in China, which has the defect of poor timeliness, and this will eventually affect the values of the calculated health losses [23,24,25,26].

**Figure 1:**
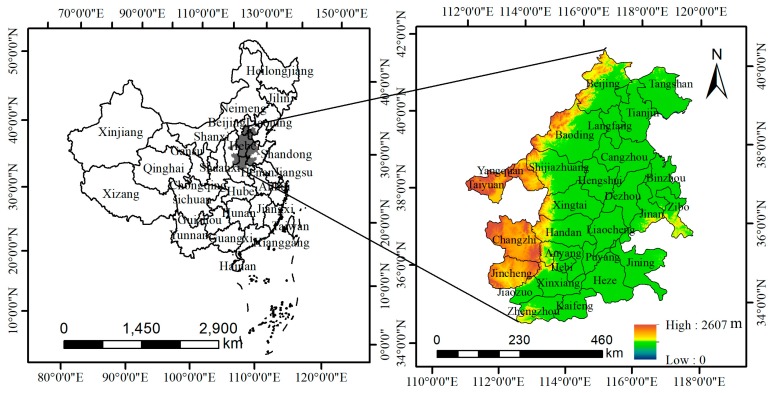
Spatial distribution of 28 cities in the study area.

Work plan on atmospheric pollution prevention and control in the Beijing–Tianjin–Hebei region and surrounding areas issued by the Ministry of Environmental Protection of China and relevant departments in 2017 indicated that Beijing, Tianjin, Shijiazhuang, Tangshan, Langfang, Baoding, Cangzhou, Hengshui, Xingtai, Handan, Taiyuan, Yangquan, Changzhi, Jincheng, Jinan, Zibo, Jining, Dezhou, Liaocheng, Binzhou, Heze, Zhengzhou, Kaifeng, Anyang, Hebi, Xinxiang, Jiaozuo, and Puyang collectively constituted the atmospheric pollution transmission channel in the Beijing–Tianjin–Hebei region, which was identified as a key area for the prevention and control of atmospheric pollution (Figure 1). According to data released by the Chinese City Statistical Yearbook, the population density of the region in 2016 was 701 persons/km^2^, while the national average population density was 144 persons/km^2^, so the region is a densely populated area in China [27]. However, the PM_2.5_ pollution in this region was extremely severe, with an average PM_2.5_ concentration of 71 μg/m^3^ in 2016, which was 7 times the guideline value issued by the World Health Organization, and posed a serious threat to the residents’ health [28]. Therefore, this paper uses the meta-analysis method and the Poisson regression model to estimate the number of deaths, hospitalizations, and outpatient emergency visits caused by PM_2.5_ pollution in cities of atmospheric pollution transmission channel in the Beijing–Tianjin–Hebei region in 2016 based on the data from literature, remote sensing data, statistical data, and survey data. Besides, the environmental value evaluation method is used to calculate their economic value, which is beneficial to provide a reference for relevant departments that formulate environmental and health policy.

## 2. Data and Methods

### 2.1. Research Framework

**Figure 2:**
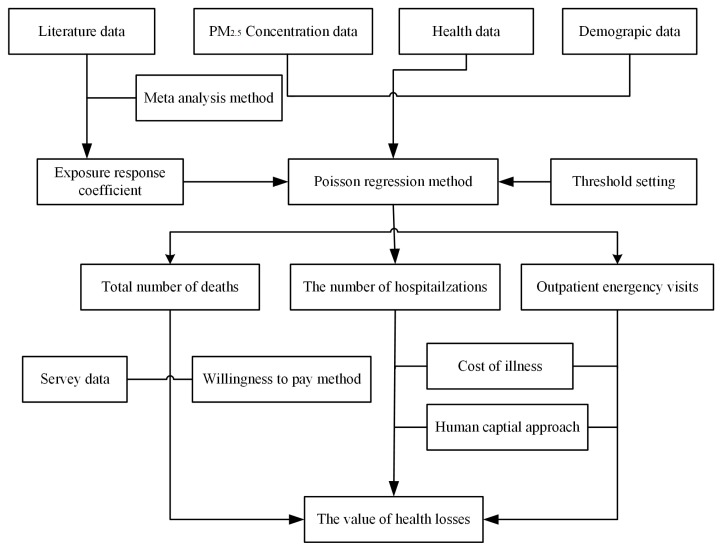
Research framework constructed in the paper.

Firstly, this paper determined the exposure–response coefficients between PM_2.5_ pollution and different health endpoints of residents by means of meta-analysis based on the collected literature data. On this basis, after combining PM_2.5_ concentration data, population data, and health data, the Poisson regression model was used to calculate the number of deaths, hospitalizations, and outpatient emergency visits induced by PM_2.5_ pollution in cities of atmospheric pollution transmission channel in the Beijing–Tianjin–Hebei region. Secondly, according to the questionnaire survey data, the willingness-to-pay method was adopted to determine the statistical life value as the unit economic value of death loss. Then, by multiplying by the number of deaths caused by PM_2.5_ pollution, the economic value of the number of deaths was calculated. Thirdly, the direct and indirect unit economic value of residents’ hospitalization and outpatient emergency visits was determined by using the disease cost method and human capital method combined with residents’ health data. The economic value of hospitalizations and outpatient emergency visits could be obtained by multiplying it by the number of hospitalizations and outpatient emergency visits induced by PM_2.5_ pollution. Finally, the economic values of the above health losses could be summarized to obtain the total economic value of health losses induced by PM_2.5_ pollution in the study area. The frame diagram of construction is shown in Figure 2.

### 2.2. Data Sources

This paper takes the 28 cities of atmospheric pollution transmission channel in the Beijing–Tianjin–Hebei region as the basic research object, and the data are mainly composed of remotely-sensed PM_2.5_ concentration, demographic and health statistics data, and questionnaire survey data. Among them, the PM_2.5_ concentration data was obtained from the Socioeconomic Data and Applications Center at Columbia University (SEDAC) based on satellite monitoring data collected using the moderate resolution imaging spectroradiometer (MODIS) and multiangle imaging spectroradiometer (MISR) instruments, providing data on PM_2.5_ concentration at a spatial resolution of 0.01° × 0.01° [29,30]. Population and health data were obtained from the China City Statistical Yearbook and China Health and Family Planning Statistical Yearbook in 2017 [31,32]. The questionnaire survey data is from the survey on the willingness of residents to pay for reducing the health risk of atmospheric pollution conducted by the research group in Zhengzhou City from October 16, 2018 to 30 November 2018. A total of 4000 questionnaires were issued in this survey, reaching a sampling ratio of approximately four parts per 10,000. The number of questionnaires recovered was 3852, and the recovery rate reached 96.3%. After eliminating the questionnaires with omissions and wrong answers, the final number of valid questionnaires was 3577, with an effective rate of 89.4%. Table 1 shows the basic statistics of the survey samples. It is important to note that due to the limitation of data availability, this paper selects a set of health endpoints, including all-cause death, death from circulatory disease, death from respiratory disease, death from lung cancer, hospitalization for circulatory disease, hospitalization for respiratory disease, and outpatient emergency treatment. Meanwhile, outpatient emergency treatment mainly refers to medical and pediatric outpatient emergency treatment closely related to PM_2.5_ pollution [26]. In addition, for some cities where it was difficult to directly obtain residents’ health data, we obtained their health data by using the total number of deaths, hospitalizations, and outpatient emergency visits in the province and multiplied by the proportion of the city’s population to the total population of the province.

### 2.3. Research Methods

#### 2.3.1. Meta-Analysis Method

The meta-analysis method was first proposed by the British psychologist Glass, and it is mainly used to summarize and analyze the research results of the same subject with specific conditions. The purpose is to increase the sample size and increase the efficiency of the inspection, as well as effectively avoid the defects of multiple studies with different qualities and different numbers of samples commonly found in the results of traditional literature [33]. The method mainly includes the following steps: (1) Selection of relevant literatures; (2) Selection of merge statistics; (3) Heterogeneity test of literatures; (4) Summary of statistics; (5) Hypothesis testing of literature combination results.

In this paper, “Air fine particulate matter”, “Mortality”, “Respiratory mortality”, “Cardiovascular mortality”, “Lung cancer mortality”, “Hospital admission”, “Respiratory”, “Cardiovascular”, “Outpatient service”, and “Emergency department visit” were used as the subject retrieval terms, and relevant literatures included in the core database of Web of Science from 1998 to 2016 were retrieved by computer to determine the exposure–response coefficients of PM_2.5_ pollution and the risk of death, hospitalization, and outpatient emergency treatment at different health endpoints of residents. The number of studies obtained was 2254. On this basis, the primary study was screened by manual retrieval, and the exclusion criteria were as follows: (1) The included meta-analysis studies should be one of a cohort of studies, time series studies, case crossover studies, and group tracking studies. (2) The studies should clearly provide the changes of PM_2.5_ concentration corresponding to the risk changes of death, hospitalization, and outpatient emergency treatment at different health endpoints. (3) For studies that fail to directly provide the relationship between PM_2.5_ concentration and the risk of death, hospitalization, and outpatient emergency treatment, the risk ratio (RR) corresponding to the change of PM_2.5_ concentration was selected as the alternative treatment, and was converted into the risk changes of death, hospitalization, and outpatient emergency treatment according to the calculation formula of RR [34]. (4) For the study research results of repeated use of epidemiological data, only the latest research results were included in the process of meta-analysis. According to the above criteria, 91 studies were selected for the meta-analysis.

#### 2.3.2. Poisson Regression Model

The assessment of health losses usually adopted the exposure–response coefficient obtained from epidemiological studies, and calculated the amount of health losses induced by specific atmospheric pollutants through the derivation and transformation of the Poisson regression model. The formula was as follows [6,17]:

(1) $$I=I_{0}*e^{\beta (C-C_{0})}$$

(2) $$\Delta I=I-I_{0}=I*\left[1-\frac{1}{e^{\beta (C-C_{0})}}\right]$$

where *I* represents the number of deaths associated with the actual PM_2.5_ concentration *C*, *I*_0_ is the number of deaths associated with the baseline PM_2.5_ concentration *C*_0_, *β* represents the exposure–response coefficient of the PM_2.5_ concentration and a specific health endpoint, and Δ*I* indicates the number of deaths or illnesses attributed to PM_2.5_ pollution. Generally, the value of *C*_0_ is between 5.8 and 8.8 μg/m^3^ [35], and the threshold concentration used in this paper is 5.8 μg/m^3^.

#### 2.3.3. Environmental Value Assessment Method

The environmental value assessment method refers to the quantitative assessment of environmental damage or benefit by some means and was presented in monetary form. Common environmental value assessment methods included the willingness-to-pay method, the human capital method, the alternative market method, the bidding game method, and so on [36]. In this paper, the willingness-to-pay method was used to measure the unit economic value of death loss, and the disease cost method and human capital method were used to measure the unit economic value of hospitalization and outpatient emergency treatment loss. For the endpoint of death, the questionnaire in the paper was set as follows: “Assuming that the fee you pay can reduce the mortality risk of residents by 1‰ in the next few years, what is the monthly fee you are willing to pay?” As a core issue, the frequency distribution of willingness to pay for reducing the health hazard of atmospheric pollution in Zhengzhou City was obtained (see Table 2).

According to Table 2, the average value of statistical life value in Zhengzhou City in 2018 reached $80,016.32. Combined with the changes of CPI (Consumer Price Index) index in Zhengzhou City, the statistical life value in 2016 was approximately $77,443.05. Taking this value as a reference, according to the per capita disposable income level of resident in different cities, the benefit conversion method is adopted to obtain the statistical life value in other cities, so as to determine the unit economic value of death loss [26]. In terms of hospitalization and outpatient emergency treatment, the average hospitalization cost, outpatient emergency treatment cost, and per capita disposable income data of resident in the province where the city is located are taken as the basis. The average hospitalization and outpatient emergency treatment costs of each city are also calculated by virtue of the benefit conversion method based on the per capita disposable income of different cities. The average number of days of hospitalization or treatment (outpatient emergency treatment day is one day) is multiplied by it, which is taken as the basis for the direct unit economic value loss of hospitalization and outpatient emergency treatment. In addition, hospitalization and outpatient emergency treatment causes indirect loss of work. Therefore, the per capita GDP of each city divided by the total number of days per year is taken as the economic value of the day when residents miss work. This is multiplied by the number of days in hospital and outpatient emergency departments (the number of days of missed work due to hospitalization is the same as the number of days in hospital, and the number of days of missed work for outpatient emergency department is one day), which is the unit economic value of residents’ indirect loss of missed work. Based on the above methods, the unit economic values of different health endpoints of 28 cities in the study area are obtained, as shown in Table 3.

Based on the calculation results of deaths, hospitalizations, and outpatient emergency visits induced by PM_2.5_ pollution, the economic value of health losses caused by PM_2.5_ pollution can be obtained by combining the data provided in Table 3. The formula is as follows [25]:

(3) $$L=\overset{m}{\underset{i=1}{\sum }}\Delta I_{i}\times N_{i}$$

where *L* represents the economic value of health losses caused by PM_2.5_ pollution; Δ*I_i_* represents the number of deaths or patients corresponding to PM_2.5_ pollution-induced health endpoint *i*; *m* represents the number of residents’ health endpoints; and *N_i_* represents the unit economic value of health endpoint *i*.

## 3. Results and Analysis

### 3.1. Determination of Exposure–Response Coefficients

**Figure 3:**
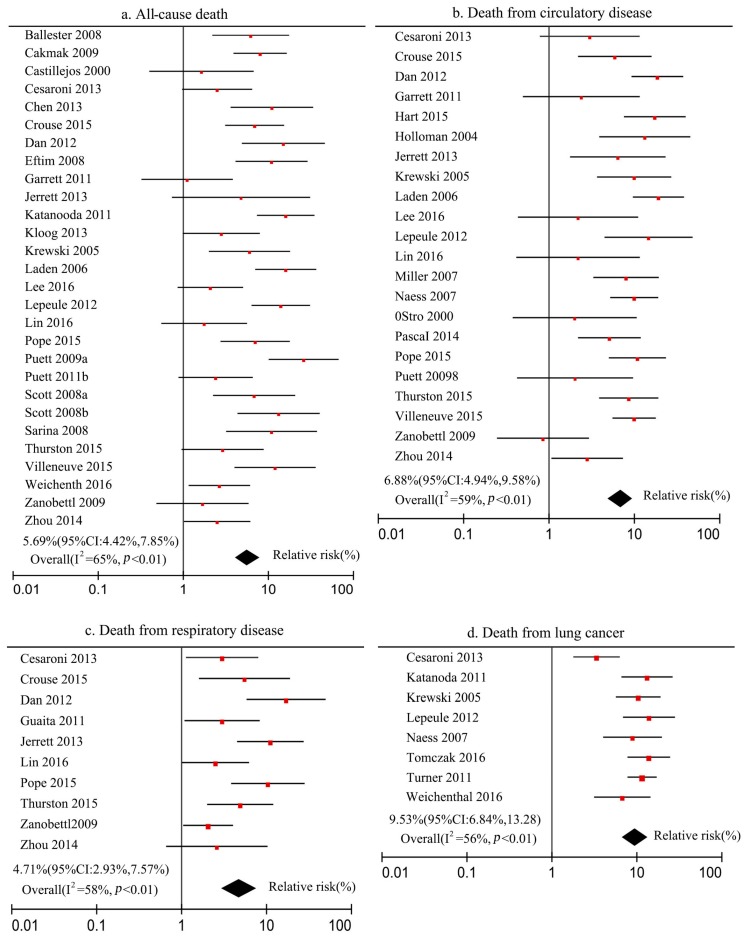
Meta-analysis of death risk at different health endpoints of residents and PM_2.5_ concentration.

The relationships between PM_2.5_ and the death risk of all-cause, circulatory disease, respiratory disease, and lung cancer were meta-analyzed using Review Manager 5.3 software (Cochrane Community, Kaifeng City, China), and the forest map was drawn (see Figure 3) based on the selected studies.

Figure 3 shows that the number of studies included in the meta-analysis were 28, 22, 10, and 8 for parts a–d, respectively. On this basis, the heterogeneity test results of studies show that the PM_2.5_ concentration and death risk of all-cause, circulatory disease, respiratory disease, and lung cancer were 65%, 59%, 58%, and 56% for parts a–d, respectively. These values were all greater than 50%, indicating that these studies have heterogeneity. Thus, the above meta-analysis process is suitable for the calculation method of the random effects model for combining the literature statistics. The combined results of the literature statistics showed that for every 10 μg/m^3^ increase in PM_2.5_ concentration, the death risk of all-cause, circulatory disease, respiratory disease, and lung cancer increased by 5.69% (95% CI: 4.12%, 7.85%), 6.88% (95% CI: 4.94%, 9.58%), 4.71% (95% CI: 2.93%, 7.57%), and 9.53% (95% CI: 6.84%, 13.28%), respectively. In addition, the P values were all less than 0.01, indicating that the hypothesis test results were very significant, which also indirectly confirmed that the increase of PM_2.5_ concentration would lead to a significant increase in the death risk of all-cause, circulatory disease, respiratory disease, and lung cancer.

**Figure 4:**
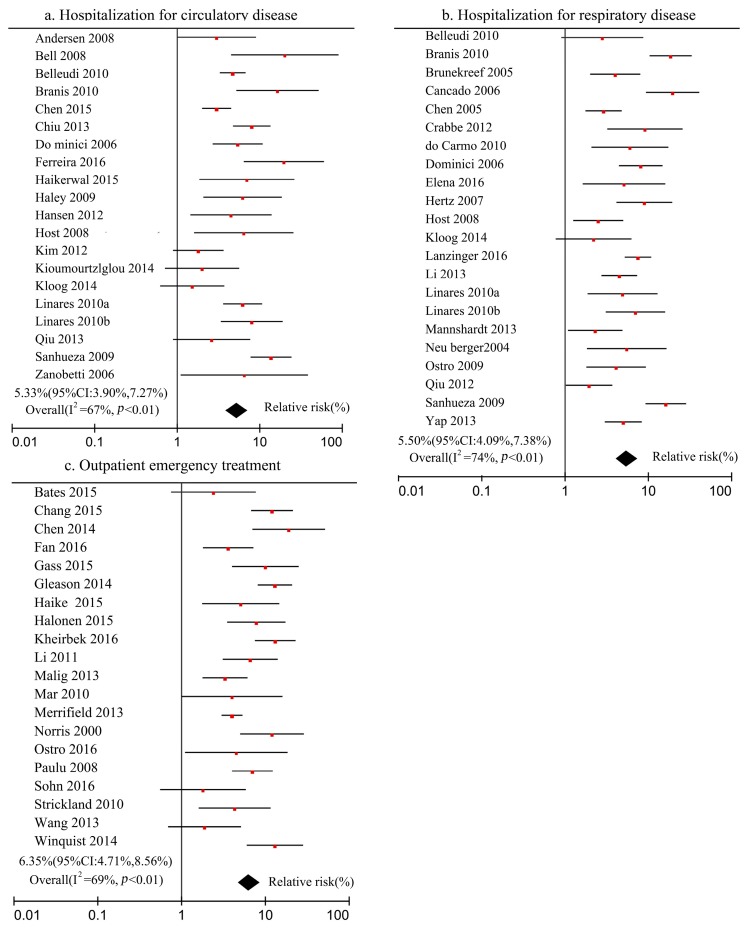
Meta-analysis of hospitalization and outpatient emergency treatment and PM_2.5_ concentration.

Similarly, the relationships between PM_2.5_ and the risk of hospitalization for circulatory, hospitalization for respiratory disease, and outpatient emergency treatment were meta-analyzed using Review Manager 5.3 software, and the forest map was shown in Figure 4.

Figure 4 shows that the number of studies included in the meta-analysis was 20, 22, and 20 for parts a–c, respectively, and the heterogeneity test values of the studies were 67%, 74% and 69%, for parts a–c, respectively. This indicates that the results have heterogeneity, so the above process of meta-analysis is suitable for the calculation method of the random effects model used to combine the literature statistics. The combined results of the literature statistics showed that every 10 μg/m^3^ increase in PM_2.5_ concentration resulted in 5.33% (95% CI: 3.90%, 7.27%), 5.50% (95% CI: 4.09%, 7.38%), and 6.35% (95% CI: 4.71%, 8.56%) increase in the risks of hospitalization for circulatory diseases, hospitalization for respiratory diseases, and outpatient emergency treatment for residents, respectively.

### 3.2. Accounting of Residents’ Health Losses

**Figure 5:**
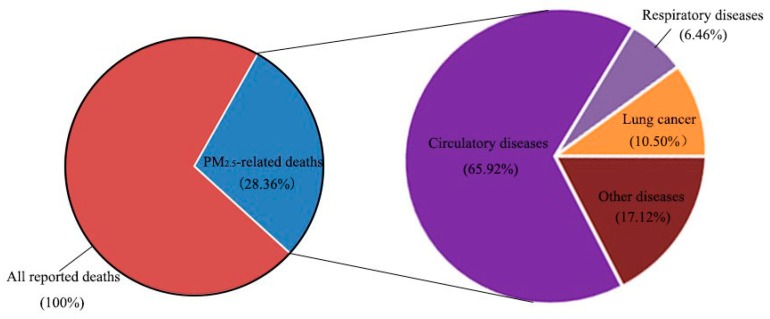
Number of deaths caused by PM_2.5_ pollution at different health endpoints in the study area.

**Figure 6:**
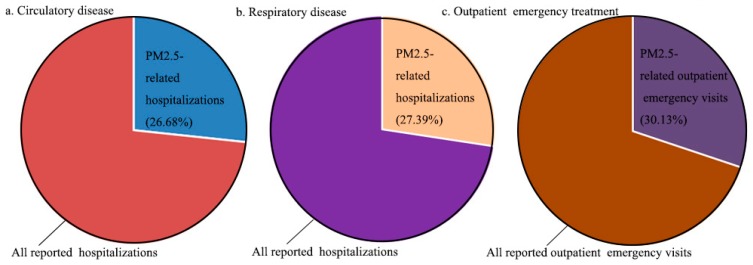
Number of hospitalizations and outpatient emergency visits induced by PM_2.5_ pollution in study area.

Based on the collected PM_2.5_ concentration data, population data, and health data, combined with the determined exposure–response coefficients, the number of deaths (see Figure 5 and Table 4), hospitalizations, and outpatient emergency visits (see Figure 6 and Table 5) induced by PM_2.5_ pollution in the study area in 2016 were calculated by using Equations (1) and (2), respectively, in order to determine the health losses of PM_2.5_ pollution.

As can be seen from Figure 5, the number of deaths in the study area in 2016 was approximately 1.092 million, and the number of deaths caused by PM_2.5_ pollution reached 309,643, accounting for 28.36% of the total number of deaths. This indicates that PM_2.5_ pollution has become an important factor in population death. Among all-cause deaths induced by PM_2.5_ pollution, the number of residents who died from circulatory disease reached 204,109, accounting for 65.92%; the number of residents who died from lung cancer was 32,518, accounting for 10.50% of the total number of PM_2.5_ pollution-induced deaths; the number of residents who died from respiratory disease was the lowest, at 20,012 persons, accounting for 6.46%. Note that the combined ratio of the three is only 82.88%, indicating that PM_2.5_ pollution may induce death at other health endpoints besides the death from circulatory disease, lung cancer, and respiratory disease, which will also be the focus of future research.

Table 4 shows that the all-cause deaths in Tianjin and Beijing were 25,045 and 28,097, respectively. The all-cause deaths in Shijiazhuang, Baoding, Cangzhou, Handan, Jinan, Liaocheng, Xingtai, Dezhou, Heze, Jining, Zhengzhou, and Xinxiang ranged from 10,000 to 20,000. Less than 10,000 persons remained in the other cities, especially for Taiyuan, Yangquan, Changzhi, Jincheng, Jiaozuo, and Hebi, with the all-deaths being less than 5000. In terms of deaths from circulatory disease, more than 12,000 persons died in Beijing, Tianjin, Shijiazhuang, Baoding, and Handan; the deaths in Cangzhou, Xingtai, Jinan, Liaocheng, Heze, Hengshui, Dezhou, Jining, Zhengzhou, and Xinxiang ranged from 6000 to 12,000; the deaths in Tangshan, Langfang, Binzhou, Zibo, Kaifeng, Jiaozuo, Anyang, Puyang, Taiyuan, Yangquan, Changzhi, Jincheng, and Hebi were below 6000 persons. In terms of deaths from respiratory disease, more than 1,500 persons died in Beijing and Tianjin; the deaths in Shijiazhuang, Baoding, Cangzhou, Handan, Jinan, Liaocheng, Zhengzhou, Xingtai, Dezhou, Heze, Jining, and Xinxiang ranged from 600 to 1200; the deaths in other cities were below 600 persons. For deaths from lung cancer, Beijing and Tianjin had the highest number of deaths, followed by Jinan, Liaocheng, and Heze; the deaths in Shijiazhuang, Baoding, Cangzhou, Xingtai, Handan, Dezhou, Jining, Zhengzhou, Tangshan, Langfang, Hengshui, Binzhou, Zibo, Kaifeng, Xinxiang, Anyang, and Puyang ranged from 500 to 1500; the deaths in other cities were below 500 persons.

As can be seen from Figure 6, the number of hospitalizations for circulatory disease in the study area in 2016 was 3,625,800, and the number of hospitalizations for circulatory disease caused by PM_2.5_ pollution reached 967,300, accounting for 26.68% of the total number of hospitalizations for circulatory disease. In the aspect of hospitalization for respiratory disease, 3,285,100 persons were admitted to the hospital, including 899,939 people exposed to PM_2.5_ pollution, accounting for 27.39% of the total number of hospitalizations for respiratory disease. In terms of outpatient emergency treatment, the statistical visits were approximately 158 million person-times, while the visits induced by PM_2.5_ pollution reached 47,655,405 person-times, accounting for 30.13%.

Table 5 shows that the hospitalizations for circulatory disease in Tianjin and Beijing were largest, both exceeding 70,000; the number of hospitalizations for circulatory disease in Shijiazhuang, Baoding, Cangzhou, Handan, Jinan, Heze, Jining, Tangshan, Xingtai, Dezhou, Liaocheng, and Zhengzhou ranged from 30,000 to 60,000; the number of hospitalizations for circulatory disease in other cities were less than 30,000, especially in Taiyuan, Yangquan, Changzhi, Jincheng, and Hebi, where the number of hospitalizations remained below 15,000. In terms of hospitalization for respiratory disease, more than 60,000 persons were hospitalized in Beijing and Tianjin; the hospitalizations in Shijiazhuang, Baoding, Handan, Jining, and Heze ranged from 45,000 to 60,000; the cities with 30,000 to 45,000 hospitalizations included Tangshan, Cangzhou, Xingtai, Jinan, Dezhou, Liaocheng, and Zhengzhou; the cities with 15,000 to 30,000 hospitalizations included Langfang, Hengshui, Binzhou, Zobo, Kaifeng, Xinxiang, Anyang, and Puyang; Taiyuan, Yangquan, Changzhi, Jincheng, Jiaozuo, and Hebi all had fewer than 15,000 residents hospitalized. For outpatient emergency treatment, more than 9.7 million person-times were made in Beijing and Tianjin; the outpatient emergency visits in Baoding, Shijiazhuang, Tangshan, Cangzhou, Xingtai, Handan, Jinan, Dezhou, Liaocheng, Heze, Jining, and Zhengzhou were also relatively high, between 1.2 million and 2.4 million person-times; the number of visits in Langfang, Hengshui, Binzhou, Zibo, Kaifeng, Xinxiang, Anyang, Puyang, Taiyuan, Yangquan, Changzhi, Jincheng, and Hebi had fewer than 1.2 million visits.

### 3.3. Value Assessment of Residents’ Health Losses

On the basis of measuring health losses of residents induced by PM_2.5_ pollution, and combining with the unit economic values of different health endpoints determined in Table 3, Formula (3) can be used to calculate the economic values of health losses induced by PM_2.5_ pollution of different cities in the study area in 2016 (see Table 6).

It can be found from Table 6 that the economic value of health losses induced by PM_2.5_ pollution in the study area in 2016 was $28.1 billion, accounting for 1.52% of the region’s GDP. From the perspective of health endpoints, the economic value of death loss was the highest, reaching $21.2 billion, and accounting for approximately 75.44% of the value of residents’ health losses. The economic value of outpatient emergency treatment loss was also relatively high, reaching $3.66 billion, accounting for 13.02%. The economic value of hospitalization loss of circulatory disease and respiratory disease was $1.68 and $1.56 billion, respectively accounting for 5.98% and 5.56%, respectively, of the value of health losses. Specifically, the economic value of health losses in Beijing and Tianjin reached $5.624 and $4.154 billion, respectively, accounting for 1.46% and 1.54% of their GDP, respectively, in the same year. The values of health losses in Jinan, Shijiazhuang, Zhengzhou, Handan, Baoding, and Cangzhou reached $1.751, $1.420, $1.241, $1.191 $1.107, and $1.095 billion, respectively. The cities with values of health losses between $600 million and $900 million included Tangshan, Langfang, Xingtai, Zibo, Jining, Dezhou, Liaocheng, Heze, and Xinxiang. The value of health losses in Hengshui, Binzhou, Kaifeng, Anyang, Jiaozuo, and Puyang ranged from $300 million to $600 million. The health losses in Taiyuan, Yangquan, Changzhi, Jincheng, and Hebi were worth less than $300 million. In conclusion, the regions with high values of residents’ health losses were distributed in large- and medium-sized cities, which had large populations and generated a strong demand for fossil energy. Therefore, PM_2.5_ pollution was serious, and the huge populations and severe PM_2.5_ pollution determined the high value of health losses in such cities.

## 4. Discussion

On the basis of determining the exposure–response coefficients of PM_2.5_ concentration and different health endpoints, this paper calculated the residents’ health losses and their economic value caused by PM_2.5_ pollution in cities of atmospheric pollution transmission channel in the Beijing–Tianjin–Hebei region in 2016. This study has important reference significance for clarifying the health hazard of PM_2.5_ pollution, formulating the prevention strategy of atmospheric pollution scientifically, and implementing the health strategy of China in depth. However, due to the availability of data, the health endpoints selected in this study were limited to all-cause death, death from circulatory disease, death from respiratory disease, death from lung cancer, hospitalization for circulatory disease, hospitalizations for respiratory disease, outpatient emergency treatment, and so on, with the defect of ignoring other health endpoints, which would underestimate the residents’ health losses caused by PM_2.5_ pollution, thus causing uncertainty in the evaluation results [37,38]. Moreover, the environmental value evaluation method was adopted to define the unit economic value of residents’ death, hospitalization, and outpatient emergency treatment loss. Similarly, due to the limitation of data availability, the paper took the statistical life value in Zhengzhou City and health data at the provincial scale as a reference, and combined the annual disposable income status of different cities, the benefit conversion method was used to calculate the unit economic value of death, hospitalization, and outpatient emergency treatment loss of different cities in the study area, as well as to calculate the economic value of health losses induced by PM_2.5_ pollution [39]. In fact, the unit economic value of loss at different health endpoints for each city was not only affected by income level, but also by population distribution, age structure, medical conditions, social security, and other factors. Therefore, there was uncertainty in the unit economic value of death, hospitalization, and outpatient emergency treatment loss calculated according to per capita disposable income, which directly determines the value assessment result of health losses. In addition, the value assessment of health losses caused by PM_2.5_ pollution based on the city as the basic research unit did not take the spatial differentiation of PM_2.5_ concentration and population distribution within the city into account. In reality, due to the differences in meteorological conditions, topographical elevation, vegetation cover, economic development, and population distribution in different regions of the city, there were major differences in the health losses suffered by residents within the city. Therefore, the question of how to carry out the value assessment of health losses on a more detailed scale had important practical significance for identifying the distribution of residents’ health risk areas.

## 5. Conclusions

(1) There were significant exposure–response relationships between PM_2.5_ pollution and health endpoints such as all-cause death, hospitalization for circulatory disease, hospitalization for respiratory disease, and outpatient emergency treatment. Among them, the increase of PM_2.5_ concentration had the greatest impact on residents’ outpatient emergency visits, followed by all-cause death and hospitalization for respiratory disease, and it had the least impact on hospitalization for circulatory disease.

(2) PM_2.5_ pollution has become an important factor inducing the death or morbidity of the population in cities of atmospheric pollution transmission channel in the Beijing–Tianjin–Hebei region. In 2016, the number of deaths, hospitalizations, and outpatient emergency visits induced by PM_2.5_ pollution in the study area reached 309,643, 1,867,240, and 47,655,405, respectively, accounting for 28.36%, 27.02% and 30.13% of the total number of deaths, hospitalizations and outpatient emergency visits in the region, respectively.

(3) The economic value of health losses caused by PM_2.5_ pollution in cities of atmospheric pollution transmission channel in the Beijing–Tianjin–Hebei region was approximately $28.1 billion, accounting for 1.52% of the GDP in the study area. The values of health losses for residents in Beijing, Tianjin, Shijiazhuang, Zhengzhou, Handan, Baoding, and Cangzhou were higher, whereas the values of health losses in Taiyuan, Yangquan, Changzhi, Jincheng, and Hebi were lower.

## Figures and Tables

**Table 1:** Basic statistics of survey samples.

Variable	Option	Proportion	Variable	Option	Proportion
Gender	Man	49.5%	Daily outdoor time	<2 h	26.0%
	Woman	50.5%		2–4 h	32.8%
Age	Youth (≤44)	36.6%		4–6 h	18.7%
	Middle (45–59)	39.6%		>6 h	22.5%
	Old (≥60)	23.8%	Health condition	Very good	28.2%
Education	≤Middle school	24.7%		Good	41.3%
	High school	29.0%		General	26.7%
	Junior college	23.5%		Poor	3.1%
	Undergraduate	19.4%		Very poor	0.7%
	Postgraduate	3.4%	Possibility of living in Zhengzhou city	Very high	64.3%
Monthly income	<$453	27.1%		High	22.1%
	$453–$906	41.4%		General	6.7%
	$906–$1360	17.6%		Small	0.6%
	$1360–$1813	10.7%		Very small	1.5%
	>$1813	3.2%		Uncertainty	4.8%

Note: High school also includes technical secondary school.

**Table 2:** Frequency distribution of payment intention of residents in Zhengzhou City.

Payment Interval (dollars/month)	Annual Payment Currency (dollars)	Number of Residents (persons)	Statistical Life Value (dollars)	Proportion (%)
0	0	1053	0	29.44
0–3.02	18.13	403	730.80	11.27
3.02–6.04	54.40	450	2448.09	12.58
6.04–9.07	90.67	428	3880.68	11.97
9.07–12.09	126.94	411	5217.15	11.49
12.09–15.11	163.21	412	6724.09	11.52
15.11–18.13	199.47	229	4567.96	6.40
18.13–21.16	235.74	115	2711.03	3.21
21.16–24.18	272.01	29	788.83	0.81
24.18–27.20	308.28	22	678.21	0.62
27.20–30.22	344.55	16	551.27	0.45
>30.22	362.68	9	326.41	0.25

Note: (1) Annual payment currency = the median of the payment interval group × 12; (2) because the “>30.22” payment interval cannot obtain the group median value, the group lower limit value is used as the alternative treatment; (3) statistical life value [24] = annual payment currency × number of residents in different payment interval × 1000; (4) In 2018, 1 dollar = 6.6174 yuan.

**Table 3:** Unit economic value of health endpoint for different cities in the study area.

City	SLV (10^4^ dollar/person)	HC (dollar/person)	OETC (dollar/person-time)	City	SLV (10^4^ dollar/person)	HC (dollar/person)	OETC (dollar/person-time)
Beijing	14.42	3108.59	69.28	Jinan	9.31	1849.57	49.85
Tianjin	10.19	2361.37	45.06	Zibo	8.09	1607.00	43.30
Shijiazhuang	6.22	1347.47	37.16	Jining	6.00	1192.03	32.13
Tangshan	7.01	1518.92	41.88	Dezhou	4.80	952.74	25.67
Langfang	6.88	1491.31	41.12	Liaocheng	4.56	905.83	24.40
Baoding	4.89	1058.97	29.21	Binzhou	6.21	1232.89	33.23
Cangzhou	5.36	1161.22	32.02	Heze	4.30	854.24	23.02
Hengshui	4.47	969.38	26.72	Zhengzhou	7.70	1621.73	39.41
Xingtai	4.48	970.81	26.77	Kaifeng	4.56	960.51	23.35
Handan	5.46	1183.05	32.62	Anyang	5.31	1117.49	27.16
Taiyuan	7.46	1731.92	51.04	Hebi	5.57	1173.42	28.51
Yangquan	6.03	1399.29	41.24	Xinxiang	5.25	1105.58	26.87
Changzhi	5.25	1218.61	35.91	Jiaozuo	5.75	1211.25	29.43
Jincheng	5.65	1311.76	38.66	Puyang	4.51	950.51	23.09

Note: (1) SLV represents the statistical life value; (2) HC represents the hospitalization cost; (3) OETC represents the outpatient emergency treatment cost; (4) 6.6423 yuan could be converted into 1 dollar in 2016.

**Table 4:** Number of deaths caused by PM_2.5_ pollution of different cities in the study area.

City	All-Cause Death (persons)	Death from Circulatory Disease (persons)	Death from Respiratory Disease (persons)	Death from Lung Cancer (persons)
Beijing	25,045	13,461	2076	4767
Tianjin	28,097	16,942	1733	3989
Shijiazhuang	18,657	12,452	1104	1342
Tangshan	8748	5883	519	608
Langfang	8651	5740	511	599
Baoding	18,557	12,384	1092	1332
Cangzhou	16,725	11,031	996	1163
Hengshui	9778	6445	580	681
Xingtai	14,777	9811	880	1039
Handan	18,154	12,067	1084	1291
Taiyuan	2114	1143	205	354
Yangquan	846	503	50	102
Changzhi	2647	1318	159	317
Jincheng	1885	1118	114	227
Jinan	15,256	11,480	999	1869
Zibo	7305	5520	483	912
Jining	10,632	8033	688	1319
Dezhou	10,785	8086	717	1314
Liaocheng	15,336	11,516	1008	1845
Binzhou	6873	5181	448	836
Heze	13,463	10,200	889	1682
Zhengzhou	12,583	7051	1116	1031
Kaifeng	7947	4983	473	731
Anyang	8935	5607	531	817
Hebi	2592	1626	156	235
Xinxiang	10,517	6591	631	963
Jiaozuo	4813	3041	287	449
Puyang	7925	4946	483	704

**Table 5:** Number of hospitalizations and outpatient emergency visits caused by PM_2.5_ pollution of different cities in the study area.

City	Hospitalization for Circulatory Disease (persons)	Hospitalization for Respiratory Disease (persons)	Outpatient Emergency Visit (person-time)
Beijing	96,722	90,112	11,327,305
Tianjin	72,583	67,478	9,660,363
Shijiazhuang	51,777	48,187	1,732,705
Tangshan	36,976	34,409	1,237,717
Langfang	26,262	24,433	873,788
Baoding	54,978	51,167	1,840,382
Cangzhou	46,493	43,199	1,541,968
Hengshui	28,314	26,294	937,885
Xingtai	39,394	36,630	1,313,009
Handan	48,588	45,191	1,622,251
Taiyuan	7478	6972	235,741
Yangquan	2432	2263	76,676
Changzhi	6428	5998	202,697
Jincheng	4727	4421	148,945
Jinan	46,759	43,492	1,530,678
Zibo	27,169	25,281	892,821
Jining	49,927	46,409	1,638,365
Dezhou	40,210	37,371	1,312,459
Liaocheng	41,862	38,897	1,365,983
Binzhou	24,011	22,331	786,991
Heze	48,835	45,439	1,605,797
Zhengzhou	42,746	39,803	1,496,117
Kaifeng	22,861	21,294	797,537
Anyang	26,297	24,482	916,369
Hebi	8491	7890	295,713
Xinxiang	28,655	26,685	999,651
Jiaozuo	14,949	13,910	523,525
Puyang	21,377	19,901	741,967

**Table 6:** Economic value of health losses in different cities.

City	All-Cause Death (10^8^ dollars)	Hospitalizations for Circulatory Disease (10^8^ dollars)	Hospitalizations for Respiratory Disease (10^8^ dollars)	Outpatient Emergency Treatment (10^8^ dollars)	Values of Health Losses (10^8^ dollars)	Percentage of GDP (%)
Beijing	36.13	3.50	3.26	13.36	56.24	1.46
Tianjin	28.63	2.07	1.92	8.92	41.54	1.54
Shijiazhuang	11.61	0.80	0.75	1.04	14.20	1.59
Tangshan	6.13	0.67	0.62	0.93	8.36	0.87
Langfang	5.95	0.45	0.42	0.57	7.39	1.81
Baoding	9.07	0.64	0.60	0.76	11.07	2.12
Cangzhou	8.96	0.62	0.58	0.79	10.95	2.05
Hengshui	4.37	0.31	0.29	0.37	5.34	2.50
Xingtai	6.62	0.42	0.39	0.50	7.93	2.67
Handan	9.91	0.64	0.59	0.76	11.91	2.37
Taiyuan	1.58	0.15	0.14	0.19	2.06	0.46
Yangquan	0.51	0.04	0.04	0.05	0.63	0.67
Changzhi	1.39	0.09	0.08	0.10	1.67	0.87
Jincheng	1.06	0.07	0.07	0.09	1.29	0.81
Jinan	14.21	1.02	0.95	1.34	17.51	1.78
Zibo	5.91	0.53	0.49	0.73	7.67	1.15
Jining	6.38	0.69	0.64	0.87	8.59	1.33
Dezhou	5.17	0.46	0.43	0.61	6.67	1.51
Liaocheng	6.99	0.45	0.42	0.60	8.47	1.97
Binzhou	4.27	0.35	0.33	0.47	5.41	1.46
Heze	5.79	0.47	0.44	0.57	7.27	1.88
Zhengzhou	9.69	0.84	0.78	1.11	12.41	1.02
Kaifeng	3.62	0.25	0.24	0.31	4.43	1.68
Anyang	4.74	0.34	0.31	0.40	5.79	1.89
Hebi	1.44	0.12	0.11	0.14	1.81	1.56
Xinxiang	5.52	0.36	0.34	0.42	6.64	2.04
Jiaozuo	2.77	0.22	0.20	0.28	3.47	1.10
Puyang	3.58	0.24	0.22	0.29	4.33	1.98

Note: 6.6423 yuan could be converted into 1 dollar in 2016.
